# Supplementary material for: Tunable inverse spin Hall effect in nanometer-thick platinum films by ionic gating
Source: Nat Commun. 2018 Aug 7;9:3118. doi: 10.1038/s41467-018-05611-9 (PMC6081370; doi:10.1038/s41467-018-05611-9)
Supplement: Supplementary file 1 — Supplementary Information [file 41467_2018_5611_MOESM1_ESM.pdf]

Supplementary Information for the manuscript

Tunable inverse spin Hall effect in nanometer-thick platinum films by ionic gating

Dushenko *et al*.

Supplementary Note 1. Sample fabrication procedure

Supplementary Figure 1 shows step-by-step fabrication procedure of the sample. The gate electrode was located directly on top of the gel and above the Pt channel. While the polymer inside the ionic gel and the copper wire connected to the gate using silver paste provide necessary mechanical support to carry weight of the gate electrode, this procedure does require extra care and high skill of the researcher preparing the sample, since it is easy to disrupt the ionic gel during the placement of the gate. Electrically insulating double-side adhesive tape on the sides of the Pt channel can be used to provide additional mechanical support for the gate electrode film (similar to piers in beam-type bridges) and to lock it in place at the same time, as illustrated in Supplementary Figure 1. Insulating tape also helps to prevent direct physical contact between silver paste for the drain-source contacts and the ionic gel, which—depending on the exact type and amount of the silver paste, ionic liquid, polymer and dissolvent used—may lead to slow deterioration of the electrical contacts made with silver paste after prolonged periods of time.

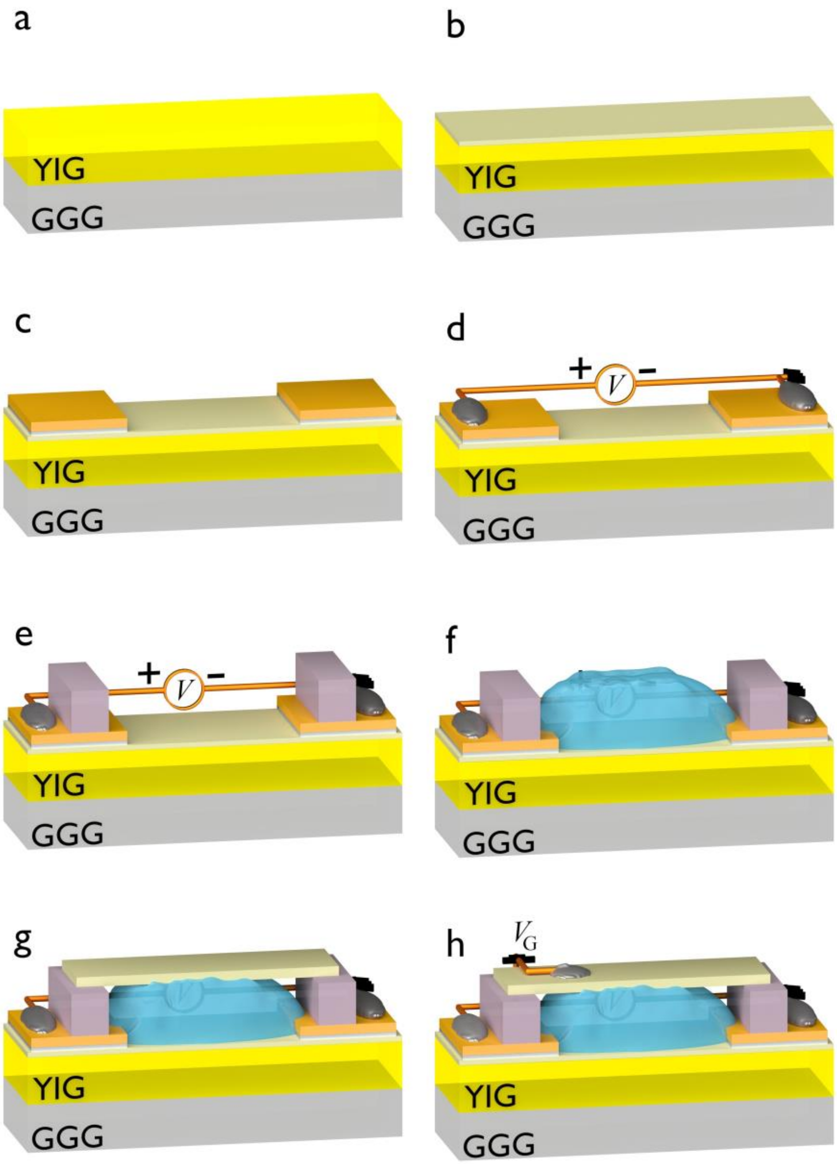

Supplementary Figure 1 | Sample fabrication procedure. a, Preparation of GGG/YIG substrate. **b**, Fabrication of Pt channel using sputtering. **c**, Evaporation of Ti/Au drain-source contact pads. **d**, Fabrication of drain-source contacts using Ag paste and Cu wires. **e**, Preparation of the mechanical support for the gate using insulating adhesive tape. **f**, Putting ionic gel on the sample. **g**, Fabrication of the top gate on the sample. **h**, Preparation of the gate contact using Ag paste and Cu wires.

Supplementary Note 2. Schematics of the measurement procedure

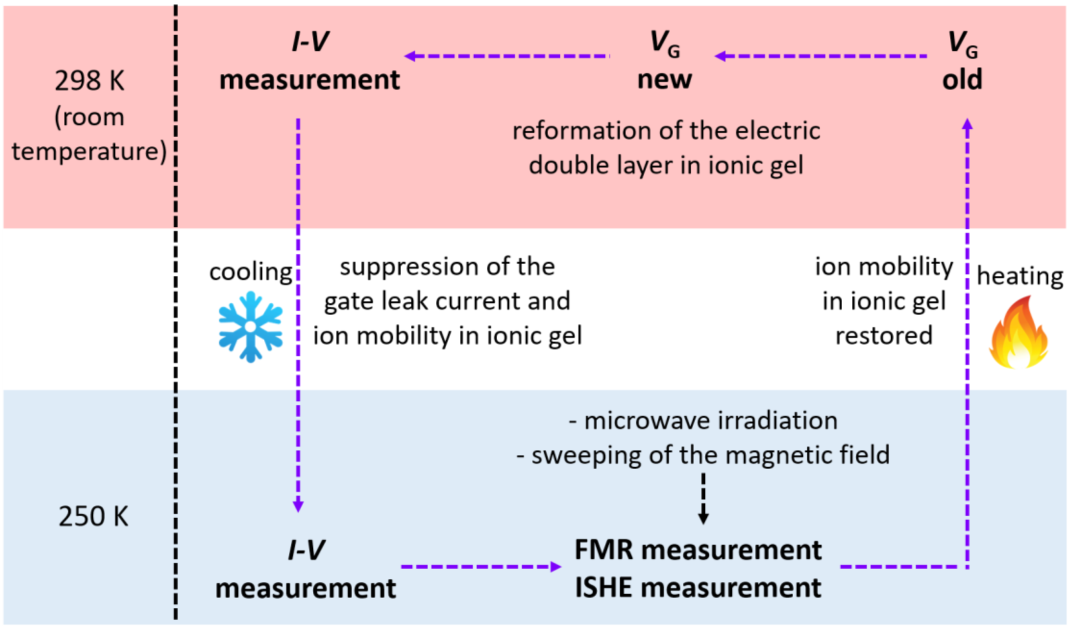

Supplementary Figure 2 | Measurement procedure. Schematics of the spin pumping and spin-charge conversion measurement procedure. Purple dashed arrows indicate order in which measurements were carried out.

Supplementary Note 3. Thickness dependence of the resistivity for thin Pt films

Figure 1 of the Main Text shows thickness dependence of the resistivity measured from the samples with d_{Pt} in the range 1.5 nm - 20 nm. Note that Eq. 23 from the literature[1] is accurate in the limit $d_{Pt}/\lambda_{mfp} > 0.1$. Thus, for Pt thickness close to 1 nm it may cause difference between the fitting curves and experimental data. Thin Pt layers can exhibit oscillations of the resistivity due to the quantum size effect. However, we can rule out such effects because of the thermal broadening at room temperature and root mean square surface roughness on the order of 0.3-0.4 nm. Fischer et al. showed that quantum size effect can only be observed for the Pt samples with surface roughness below 0.3 nm.[2] For samples with larger roughness it becomes comparable to or exceeds Fermi wavelength of the carriers, which averages out the quantum size effect of thickness.

Supplementary Note 4. Mechanism of the resistivity modulation using ionic gel

Ionic liquid is basically a salt in liquid form: it consists of mixture of the positive and negative molecules. Under the applied gate voltage, molecules in the ionic liquid form charged layer at the channel interface that acts as a capacitor with very small separation distance. In our study, we used an ionic gel: mixture of the DEME-TFSI ionic liquid with the PS-PMMA-PS polymer. Polymer was introduced to increase the viscosity of the ionic liquid to help it retain shape under prolonged periods of time (up to days). While commonly it is assumed that carrier modulation is electrically induced through the capacitance of the ionic liquid, recent studies showed that electrochemical effects due to water molecules dissolved into H^+ and O^- ions can also provide significant contribution.[3] One can separate capacitance-induced from electrochemically-induced carrier modulation by controlling number of the water molecules present in the system.[4,5] We observe gate modulation of resistivity in both air and nitrogen atmosphere, which evidences against dominant influence of H^+ and O^- ions on carrier density. Additionally, we measured *I-V* curves at various gate voltages to reveal absence/presence of the oxidized Pt layer. Figures 4o-t of the Main Text show the measured drain source current I_{DS} dependence on the drain-source voltage V_{DS} measured at various gate voltages in 2 nm Pt layer (see Supplementary Note 9 for *I-V* curves at all measured gate voltages, and examples of *I-V* curves from other measured devices). Linear *I-V* curves at both negative and positive gate voltage indicate that there is no large oxidation of Pt layer, since Pt-oxides (PtO, and α- and β-PtO_2) are semiconductors with bandgap in the range 1.3-1.8 eV [6–8] and exhibit nonlinear *I-V* characteristics.[9–11] However, we note that linear *I-V* curve alone doesn't completely rule out the possibility of some oxidation present: Abe et al. showed that transition to semiconducting from metallic behavior only happens for $x > 0.6$ in $Pt_{1-x}O_x$ alloys.[9] Finally, the oxidation effect would be largest at negative gate voltage, when negatively charge oxygen ions accumulate at the Pt interface.[3] In contrast, we observe resistivity modulation at both positive and negative gate voltage (Fig. 5a), thus, it cannot be only caused by the oxidation of the Pt layer. The slower resistivity modulation rate at large gate voltage (also observed in other studies[12]) signifies that formation of the electric double layer is finished, and electric field is saturated—meaning that larger gate voltage doesn't alternate electric double layer at the channel interface, thus doesn't lead to change in the induced carrier density. It also confirms that modulation of the resistivity with gate voltage sweep doesn't originate from the chemical reaction, for which rate would increase together with the gate voltage. We conclude that in our samples the gate voltage effect on resistivity of Pt layer can be separated into the reversible and irreversible parts. The resistance modulation shown in Fig. 5a is generated by the carrier modulation in the channel via the ionic layer capacitance effect, with the possible contribution from the reversible accumulation of the H^+ and O^- ions. Additionally, in most cases we observe the small increase in resistivity of the sample between the measurements (typically $\leq 5\%$, see Supplementary Note 5), which is probably caused by the irreversible electrochemical reaction with oxygen ions at Pt surface. More precise separation between the carrier inducing mechanisms is an ongoing research topic in the field of ionic liquid gating, and beyond the scope of this paper. We stress, however, that the resistivity modulation (and the ISHE modulation described later in the paper) is reversible and reproducible both between different measurements of the same sample and measurements of

different samples. Moreover, we did not observe any deterioration in the resistivity modulation efficiency *k* in 15 consecutive gate voltage sweeps (Supplementary Note 5).

Supplementary Note 5. Ionic gel gate characteristics

In the ionic liquids the possible asymmetry between negative and positive gate voltage can be present due to the sweep direction hysteresis effect in the ionic liquid. In the Main Text, we used only data from the positive direction of the gate voltage sweep (upsweep: from negative to positive values of gate voltage). Additional asymmetry between carrier modulation efficiency of the negative and positive gate voltage can originate from the difference in size of negatively and positively charge molecules in the ionic liquid, which leads to different spacing between charged ionic layer and channel. See Supplementary Note 10 for further discussion of the hysteresis effect on the measurements.

As discussed in the Supplementary Note 4, the gate voltage effect on resistance of Pt layer can be separated into the reversible and irreversible parts. The resistance modulation shown in Fig. 5a is generated by the carrier modulation in the channel via the ionic layer capacitance effect, with the possible contribution from the reversible accumulation of the H^+ and O^- ions. In most cases we also observe the small increase in resistivity of the sample between the separate gate voltage sweeps (typically ≤ 5%), which is probably caused by the irreversible electrochemical reaction with oxygen ions at Pt surface. Supplementary Figure 3 shows an example of the effect. After finishing the spin pumping and spin-charge conversion measurements in the range of gate voltages from -2.0 V to 2.0 V on 2.5 nm-thick Pt sample (data shown in Fig. 5c of the Main Text), we performed additional consecutive sweeps of gate voltage from -2.0 V to 2.0 V at room temperature in ambient conditions. The average increase of sample resistance at V_G = -2.0 V between consecutive sweeps of gate voltage was 2.5%. We stress, however, that the resistance modulation (and the ISHE modulation described later in the Main Text) during the gate voltage sweep itself is reversible and reproducible. Supplementary Figure 4 shows the gate efficiency (resistance modulation factor k described in the Main Text) for different gate voltage sweeps. The gate continued to function, and sample resistance was repeatedly modulated through the all sweeps.

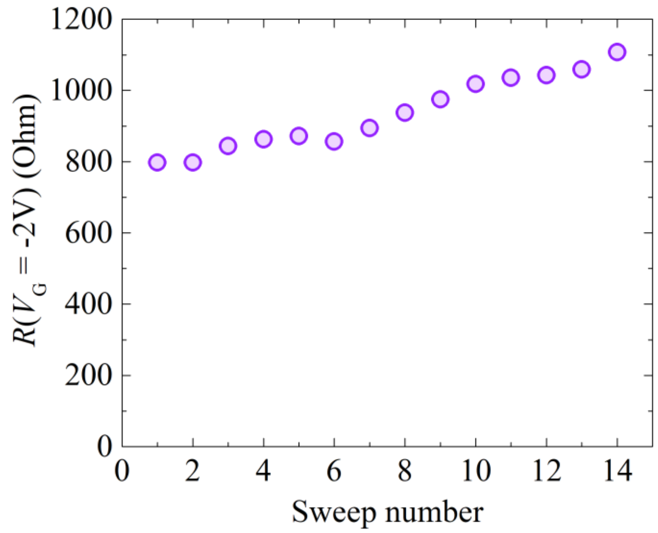

Supplementary Figure 3 | Sweep number dependence of the Pt resistance. Dependence of the sample resistance at V_G = -2 V for the consecutive sweeps of the gate voltage from -2.0 V to 2.0 V for the 2.5 nm-thick sample at room temperature in ambient conditions. Sweeps number 1-5 were upsweeps, sweeps number 6-14 were downsweeps. The average increase of the sample resistance at V_G = -2.0 V between consecutive sweeps of gate voltage was 2.5%.

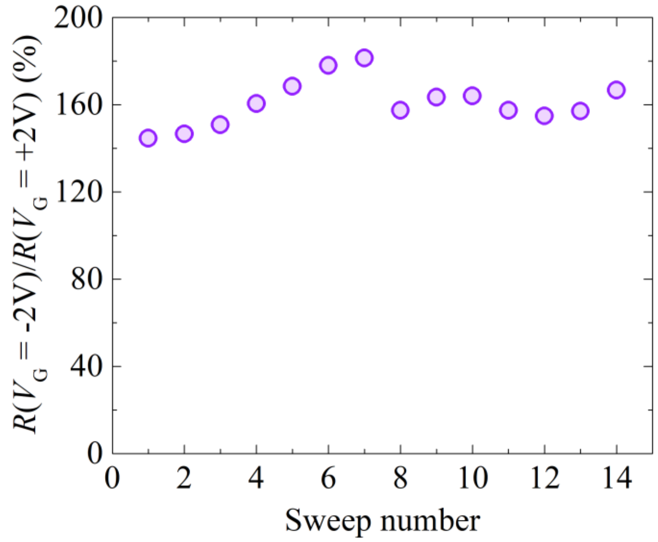

Supplementary Figure 4 | Sweep number dependence of the Pt resistivity modulation. Dependence of the sample resistance modulation by the gate for the consecutive sweeps of the gate voltage from -2.0 V to 2.0 V for the 2.5 nm-thick sample at room temperature in ambient conditions. Sweeps 1-5 were upsweeps, sweeps 6-14 were downsweeps.

Supplementary Note 6. Extracting spin-dependent component from voltage measurements

We extracted spin-dependent component of the electromotive force detected from the samples using reversal of the external magnetic field. Direction of the injected spins is reversed together with the direction of the external magnetic field $\theta_{\mathbf{H}}$, which results in the sign change of the spin-charge conversion current: $\mathbf{j}_c \propto \mathbf{j}_s \times \boldsymbol{\sigma}$. Supplementary Figure 5 shows the electromotive force generated in the sample with d_{Pt} = 10 nm under the ferromagnetic resonance conditions. Sign of the electromotive force was reversed between $\theta_{\mathbf{H}}$ = 0° (blue filled circles) and $\theta_{\mathbf{H}}$ = 180° (purple filled circles), showing that the generated voltage is dominated by the spin-charge conversion process. Subtracting electromotive force data for the opposite direction of the external magnetic field removes possible small contributions from the spurious effects independent of magnetic field (for example, Seebeck effect). Supplementary Figure 5 shows the electromotive force averaged over external magnetic field direction: $(V_{\theta_{\mathbf{H}}=0°}-V_{\theta_{\mathbf{H}}=180°})/2$.

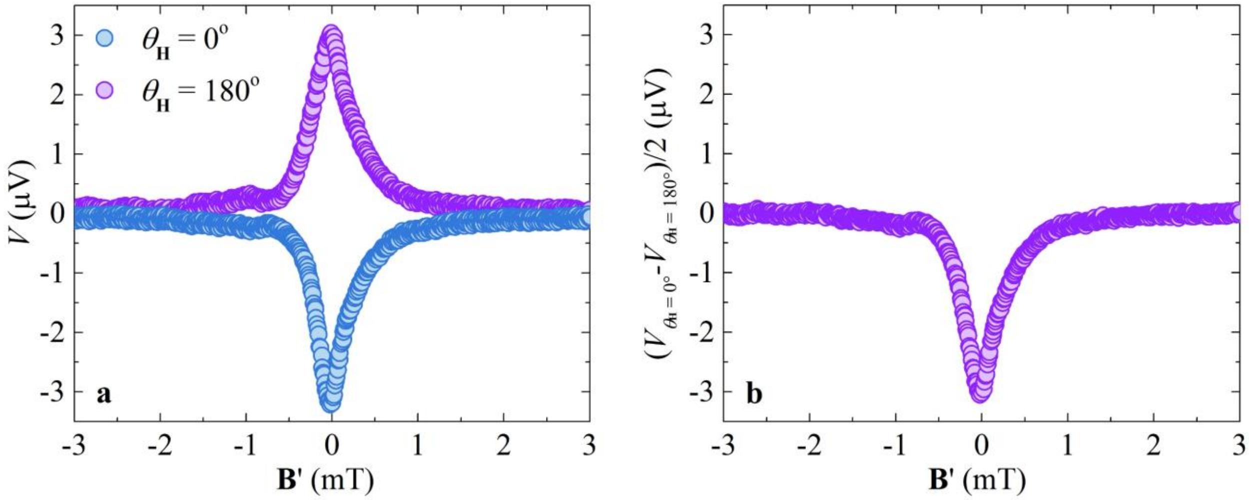

Supplementary Figure 5 | Spin-charge conversion voltage generated by spin pumping. Electromotive force measured under the ferromagnetic resonance condition with the direction of the external magnetic field $\theta_{\mathbf{H}}$ = 0° (blue filled circles) and $\theta_{\mathbf{H}}$ = 180° (purple filled circles) in the sample with d_{Pt} = 10 nm (Fig. 5c the Main Text); V_G = 0 V. **b**, Electromotive force generated in d_{Pt} = 10 nm sample at V_G = 0 V averaged over opposite directions of the external magnetic field ($\theta_{\mathbf{H}}$ = 0° and $\theta_{\mathbf{H}}$ = 180°) to remove spurious contributions; $\mathbf{B}' = \mu_0(\mathbf{H}-\mathbf{H}_{Center})$.

Supplementary Note 7. Fitting of the detected electromotive force

Electromotive force from the Pt channel during the sweeping of the external magnetic field through the ferromagnetic resonance condition of the underlying YIG layer was detected using KEITHLEY Nanovoltmeter 2182A. Supplementary Figures 6a and 7a (purple filled circles) show experimentally detected voltage averaged for the opposite directions of the external magnetic field (see Supplementary Note 6) for samples with d_{Pt} = 2 nm and d_{Pt} = 10 nm, correspondingly. Blue line shows fitting of the experimental data using the following fitting function:

$$V_{\text{Fitting}}(\mathbf{H}) = \frac{V_{\text{S1}}G_1^2}{\mu_0^2|\mathbf{H}-\mathbf{H}_{\text{FMR}}|^2+G_1^2} - \frac{2V_{\text{A1}}G_1|\mathbf{H}-\mathbf{H}_{\text{FMR}}|}{\mu_0^2|\mathbf{H}-\mathbf{H}_{\text{FMR}}|^2+G_1^2} + \frac{V_{\text{S2}}G_2^2}{\mu_0^2|\mathbf{H}-\mathbf{H}_{\text{R2}}|^2+G_2^2} + \frac{V_{\text{S3}}G_3^2}{\mu_0^2|\mathbf{H}-\mathbf{H}_{\text{R3}}|^2+G_3^2} + a\mu_0|\mathbf{H}-\mathbf{H}_{\text{FMR}}| + b, \quad (1)$$

where μ_0 is the magnetic constant (permeability of free space), V_{S1} is the dominant term that describes voltage contribution at the ferromagnetic resonance field $\mathbf{H}_{FMR}$; additional symmetric (with respect to the resonance field $\mathbf{H}_{Ri}$) terms V_{S2}, V_{S3} and asymmetrical term V_{A1} describe asymmetry in the spectrum caused by the magnetostatic surface and backward volume waves on the sides of the fundamental ferromagnetic resonance mode. Terms a and b describe contribution of the linear background. Note that the two additional symmetric peaks (V_{S2} and V_{S3} terms in the fitting function) on the sides of the main peak V_{S1} at $\mathbf{H}_{FMR}$ are generally less than 10% of V_{S1} term. Supplementary Figures 6b and 7b show deconvolution of the fitting function into the contribution from individual terms.

The ISHE voltage was calculated as value of the fitting function at the ferromagnetic resonance field $\mathbf{H}_{FMR}$ with subtracted contribution from the background:

$$V_{\text{ISHE}} = \left(V_{\text{Fitting}} - a\mu_0|\mathbf{H}-\mathbf{H}_{\text{FMR}}| - b\right)\big|_{\mathbf{H}=\mathbf{H}_{\text{FMR}}} = \\ = V_{\text{S1}} + \frac{V_{\text{S2}}G_2^2}{\mu_0^2|\mathbf{H}_{\text{FMR}}-\mathbf{H}_{\text{R2}}|^2+G_2^2} + \frac{V_{\text{S3}}G_3^2}{\mu_0^2|\mathbf{H}_{\text{FMR}}-\mathbf{H}_{\text{R2}}|^2+G_3^2} \quad (2)$$

For spectra with dominating symmetrical shape and less pronounced additional side peaks the V_{S2} and V_{S3} terms of fitting function were not used. We note that the extracted V_{ISHE} values do not change much depending on the fitting method, thus main conclusion of the paper holds true independent of the chosen method of data fitting. We confirmed that similar V_{ISHE} values are obtained using fitting function with only single symmetrical term, and also by simply measuring the maximum amplitude of the electromotive force generated during magnetic field sweep through the ferromagnetic resonance. However, the latter two methods have larger error due to the unprecise fitting and experimental noise in the voltage data, correspondingly.

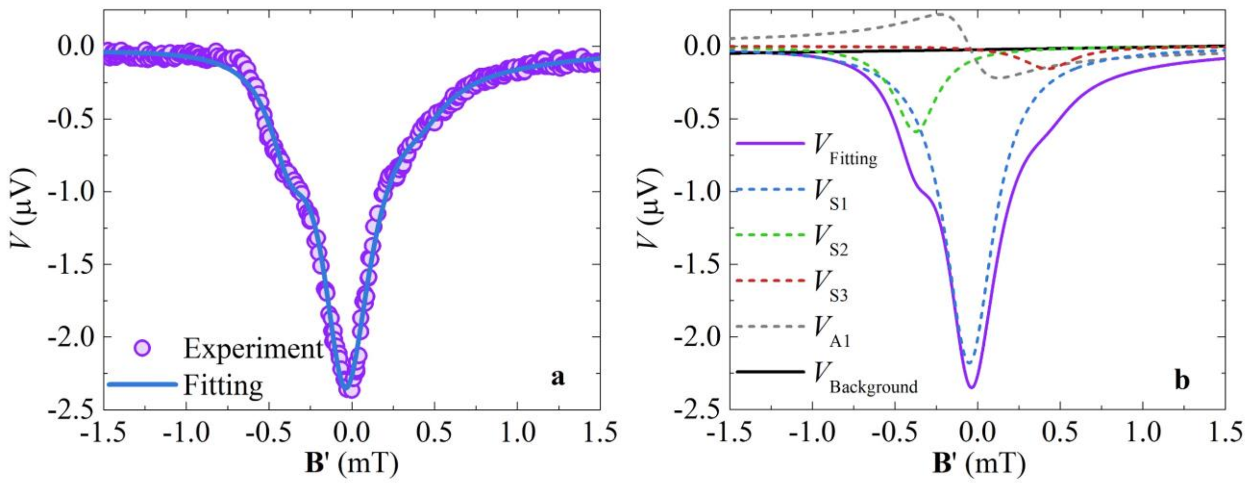

Supplementary Figure 6 | Electromotive force fitting for 2 nm-thick Pt. Fitting of the electromotive force generated in the Device A (Figs. 4 and 5 in the Main Text) with d_{Pt} = 2 nm under the ferromagnetic resonance condition and V_{G} = 0 V. **a**, Experimentally detected voltage averaged for the opposite directions of the external magnetic field (purple filled circles) and fitting of the experimental data (blue line). **b**, Deconvolution of the fitting function into the contributions from individual terms from Supplementary Equation 1: V_{Fitting}—solid purple line, V_{S1}—blue dashed line, V_{S2}—green dashed line, V_{S3}—red dashed line, V_{A1}—dashed black line, $V_{\mathrm{Background}}$—solid black line; **B**'=μ_0(**H**-**H**$_{\mathrm{Center}}$).

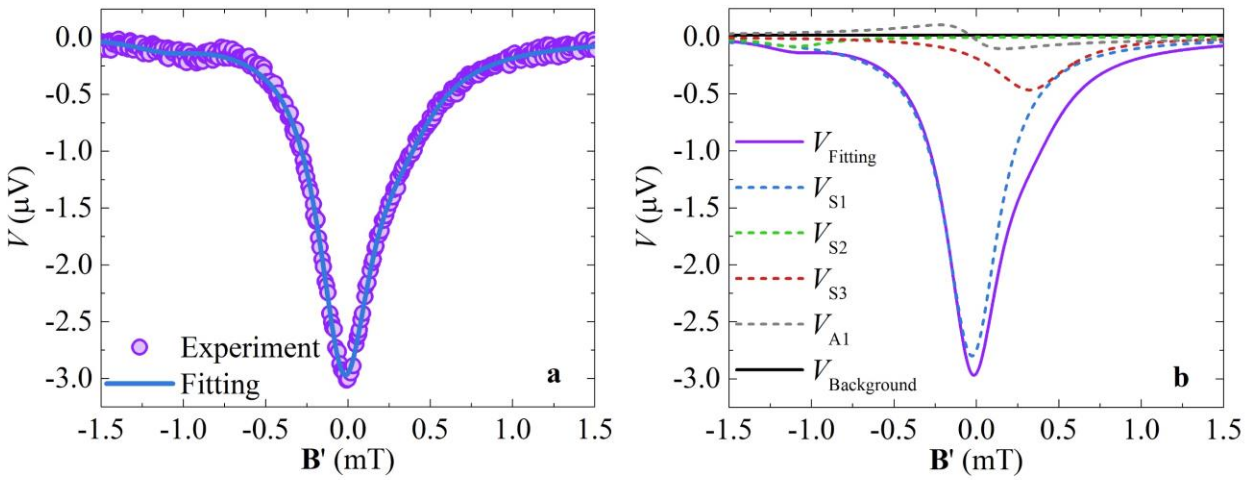

Supplementary Figure 7 | Electromotive force fitting for 10 nm-thick Pt. Fitting of the electromotive force generated in the sample with d_{Pt} = 10 nm (Fig. 5c the Main Text) under the ferromagnetic resonance condition and V_{G} = -2.0 V. **a**, Experimentally detected voltage averaged for the opposite directions of the external magnetic field (purple filled circles) and fitting of the experimental data (blue line). **b**, Deconvolution of the fitting function into the contributions from individual terms from Supplementary Equation 1: V_{Fitting}—solid purple line, V_{S1}—blue dashed line, V_{S2}—green dashed line, V_{S3}—red dashed line, V_{A1}—dashed black line, $V_{\mathrm{Background}}$—solid black line; **B**'=μ_0(**H**-**H**$_{\mathrm{Center}}$).

Supplementary Note 8. Microwave absorption spectra

Microwave absorption spectra of the samples mounted in the TE_{011} cavity of the electron spin resonance system (JEOL JES-FA200) were measured under the sweep of the in-plane external magnetic field around the ferromagnetic resonance condition of YIG layer. The applied microwave power was set to 1 mW, and the microwave frequency to f = 9.12 GHz. Supplementary Figures 8 and 9 show examples of the detected microwave absorption spectra from the samples with d_{Pt} = 2 nm (purple filled circles). Gilbert damping of the samples was extracted using fitting that consists of the combination of the Lorentzian functions and background contribution (blue solid line in Supplementary Figures 8 and 9). Details of the fitting procedure for the microwave absorption spectrum and extraction of the Gilbert damping parameter are described elsewhere.[13]

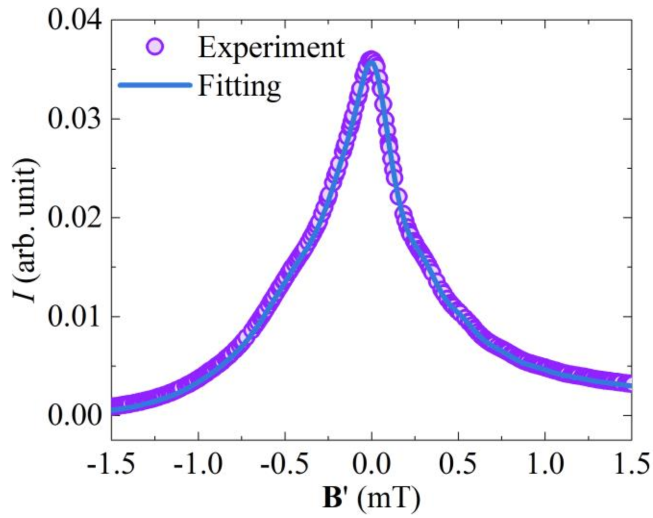

Supplementary Figure 8 | Fitting of the microwave absorption spectrum of the Device A. Experimentally measured microwave absorption spectrum of the Device A (Figs. 4 and 5 in the Main Text) with d_{Pt} = 2 nm and V_G = 0 V for the direction of the external magnetic field $\theta_{\mathbf{H}}$ = 0° (purple filled circles). Blue line shows fitting of the experimental data used to extract Gilbert damping parameter. $\mathbf{B}'=\mu_0(\mathbf{H}-\mathbf{H}_{Center})$.

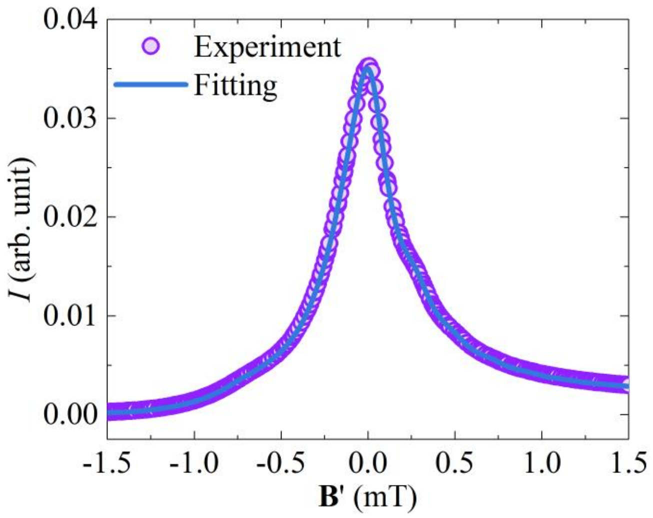

Supplementary Figure 9 | Fitting of the microwave absorption spectrum of the Device B. Experimentally measured microwave absorption spectrum of the Device B (Fig. 5 in the Main Text) with d_{Pt} = 2 nm and V_G = -2 V for the direction of the external magnetic field $\theta_{\mathbf{H}}$ = 0° (purple filled circles). Blue line shows fitting of the experimental data used to extract Gilbert damping parameter. **B**'=μ_0(**H**-**H**$_{Center}$).

Supplementary Note 9. *I*-*V* characteristics of thin Pt layers under gate voltage application

This section provides compilation of *I*-*V* curves measured for the samples with Pt channel thickness d_{Pt} = 2 nm, 2.5 nm and 10 nm under application of gate voltage. Resistance modulation efficiency (change in the slope of *I*-*V* curve) increased with decreasing thickness of the Pt channel.

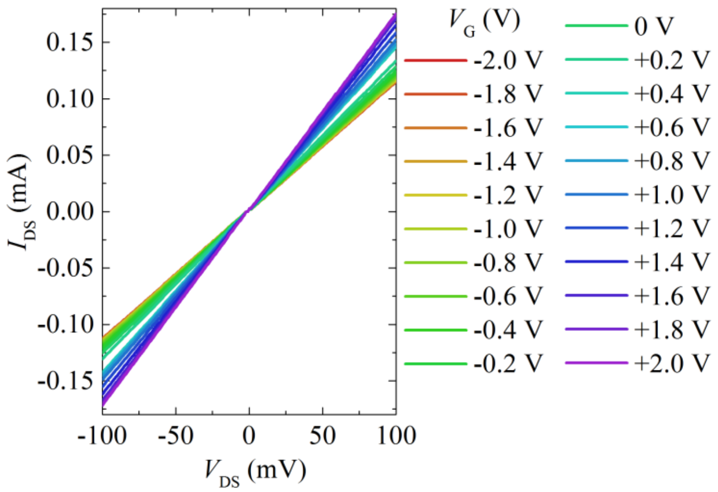

Supplementary Figure 10 | Gate dependence of *I*-*V* characteristic of the device A. Drain-source current I_{DS} dependence on the applied drain-source voltage V_{DS} at various gate voltages for the Device A with d_{Pt} = 2 nm (Fig. 5 of the Main Text) measured at 250 K.

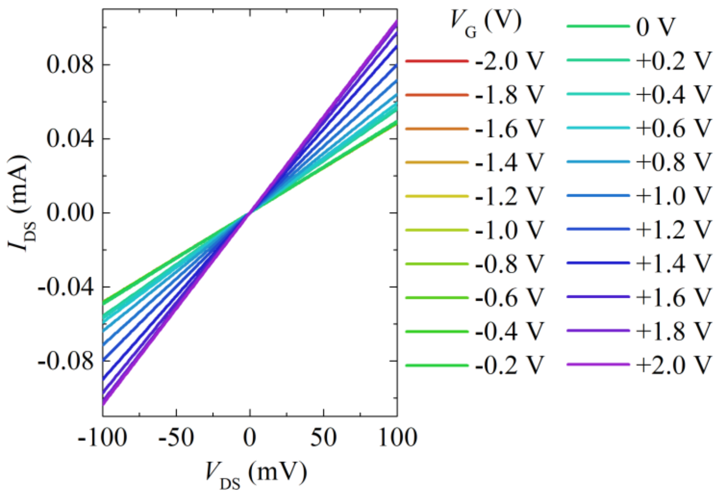

Supplementary Figure 11 | Gate dependence of *I*-*V* characteristic of the device B. Drain-source current I_{DS} dependence on the applied drain-source voltage V_{DS} at various gate voltages for the Device B with d_{Pt} = 2 nm (Fig. 5c of the Main Text) measured at 250 K.

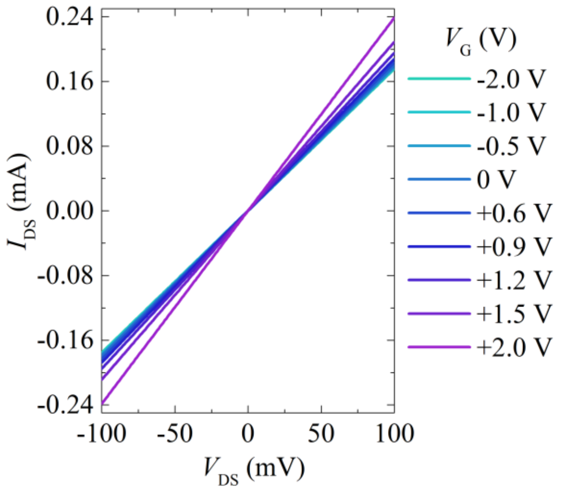

Supplementary Figure 12 | Gate dependence of *I*-*V* characteristic of 2.5 nm-thick Pt. Drain-source current I_{DS} dependence on the applied drain-source voltage V_{DS} at various gate voltages for the device with d_{Pt} = 2.5 nm (Fig. 5d of the Main Text) measured at 250 K.

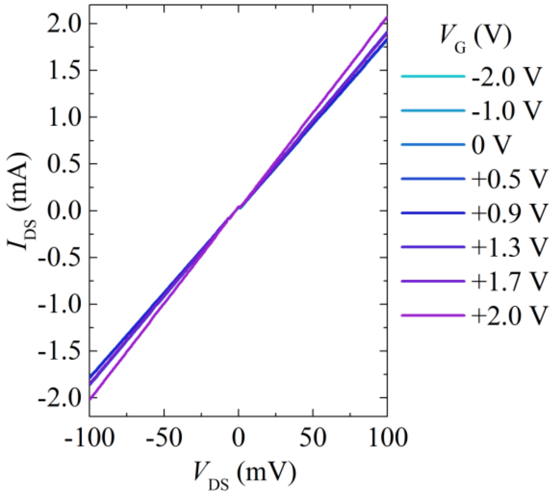

Supplementary Figure 13 | Gate dependence of *I*-*V* characteristic of 10 nm-thick Pt. Drain-source current I_{DS} dependence on the applied drain-source voltage V_{DS} at various gate voltages for the device with d_{Pt} = 10 nm (Fig. 5d of the Main Text) measured at 250 K.

Supplementary Note 10. Summary of the data for the sample d_{Pt} = 2 nm different from the one described in the Main Text.

For comparison purposes, we provide summary of the data for the d_{Pt} = 2 nm sample (Device B in Fig. 5c), different from the one described in the Main Text (Device A in Fig. 5c). Also, see *I*-*V* curves for this sample in Supplementary Figure 11.

Jump in the resistance of the sample between measurements at V_G = 0 V and V_G = 0.2 V (Supplementary Figure 14) is due to the stop of the nitrogen flow supply (refilling of the liquid nitrogen vessel). Thus, sample and ionic gel condition was changed to an extent between these two gate voltage values.

Asymmetry between negative and positive gate voltage originates from the sweep direction hysteresis effect in the ionic liquid. The development of the electric double layer in the ionic liquid for the voltage upsweep (from negative to positive V_G) and downsweep (from positive to negative V_G) has hysteresis due to the presence of the energy barriers during reorganization of the electric double layer structure[14–16] (similar to the hysteresis in magnetization curves of ferromagnets under the sweeping of a magnetic field). This hysteresis results in the shift between the up and down voltage sweeps in the gate voltage value at which the out-of-plane electric field induced at the Pt interface by the ionic gel gate changes direction. In the Main Text, we used only data from the upsweep direction of the gate voltage sweep (from negative to positive values of gate voltage). For example, most of the resistance modulation for the sample in Supplementary Figure 14 happens between -0.5 V and 2.0 V. Thus, actual switching of the direction of the electric field induced by the gate at the interface happens at V_G ~ 0.8 V.

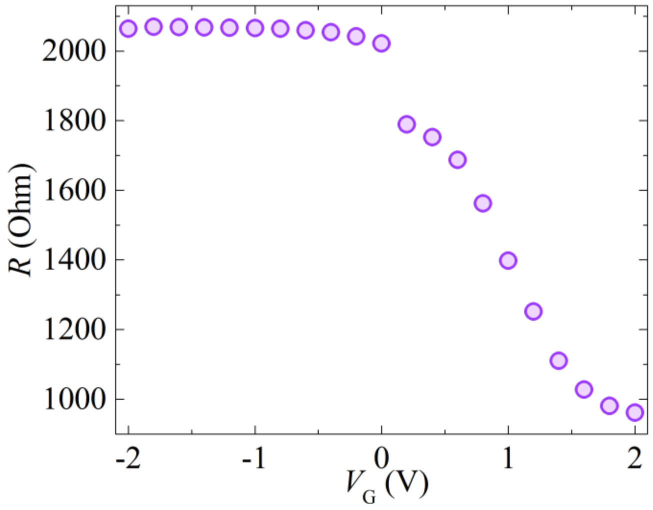

Supplementary Figure 14 | Gate dependence of the Device B resistance. Resistance modulation under application of the gate voltage V_G for the sample with Pt channel thickness d_{Pt} = 2 nm (Device B in Fig. 5c). Jump in the resistance of the sample between measurements between V_G = 0 V and V_G = 0.2 V is caused by the change in the condition of the ionic gel due to the stop of the temperature control to refill the liquid nitrogen vessel.

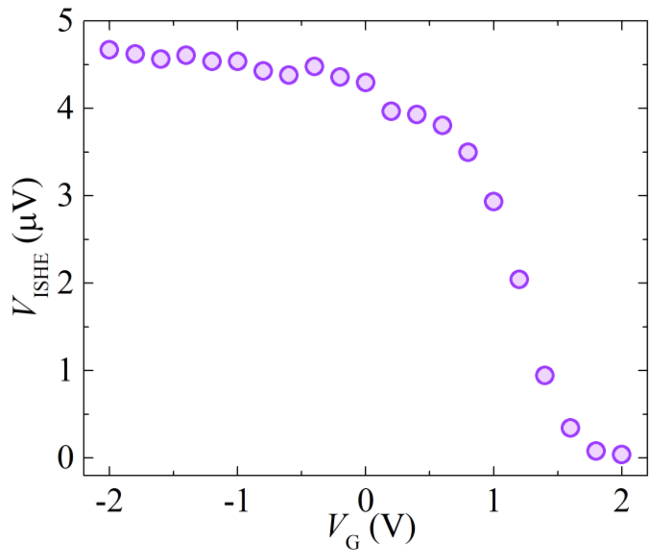

Supplementary Figure 15 | Gate dependence of the Device B electromotive force. Amplitude of the generated ISHE voltage V_{ISHE} dependence on the applied gate voltage V_G for the sample with Pt channel thickness d_{Pt} = 2 nm (Device B in Fig. 5c).

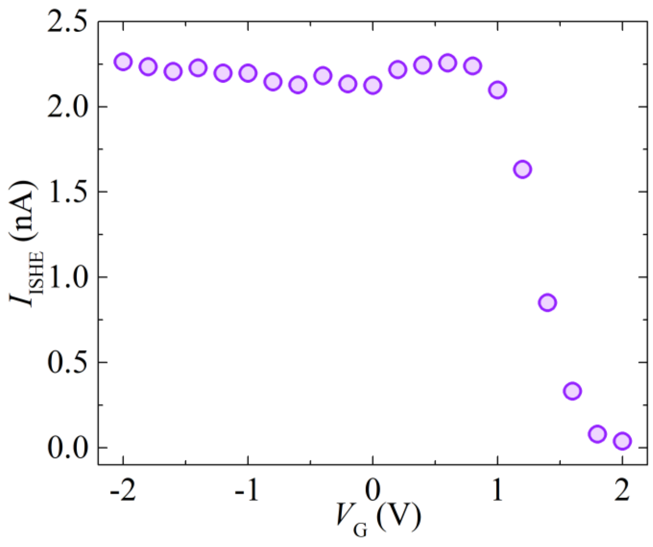

Supplementary Figure 16 | Gate dependence of the Device B spin-charge conversion current. Amplitude of the generated via the ISHE spin-charge conversion current I_{ISHE} = V_{ISHE}/R for the sample with Pt channel thickness d_{Pt} = 2 nm (Device B in Fig. 5c).

Supplementary Note 11. Atomic force microscopy

Supplementary Figure 17 shows representative atomic force microscopy images (top view) of sample with 2 nm-thick Pt channel. Supplementary Figure 18 shows profile cuts along the white lines shown in Supplementary Figure 17a-c. Difference in height of Pt film is below 1 nm, which confirms continuous character of Pt films even down to 2 nm thickness. The root mean square roughness of the samples was usually in the range 0.3-0.4 nm. It is possible to further optimize fabrication procedure by controlling the evaporation rate, and preparing even thinner devices. Fischer et al. reported inverse dependence between the sputtering rate and surface roughness[2], though high rate comes at a cost of higher energy of sputtered atoms, which can damage YIG surface and decrease spin mixing conductance at the YIG/Pt interface.

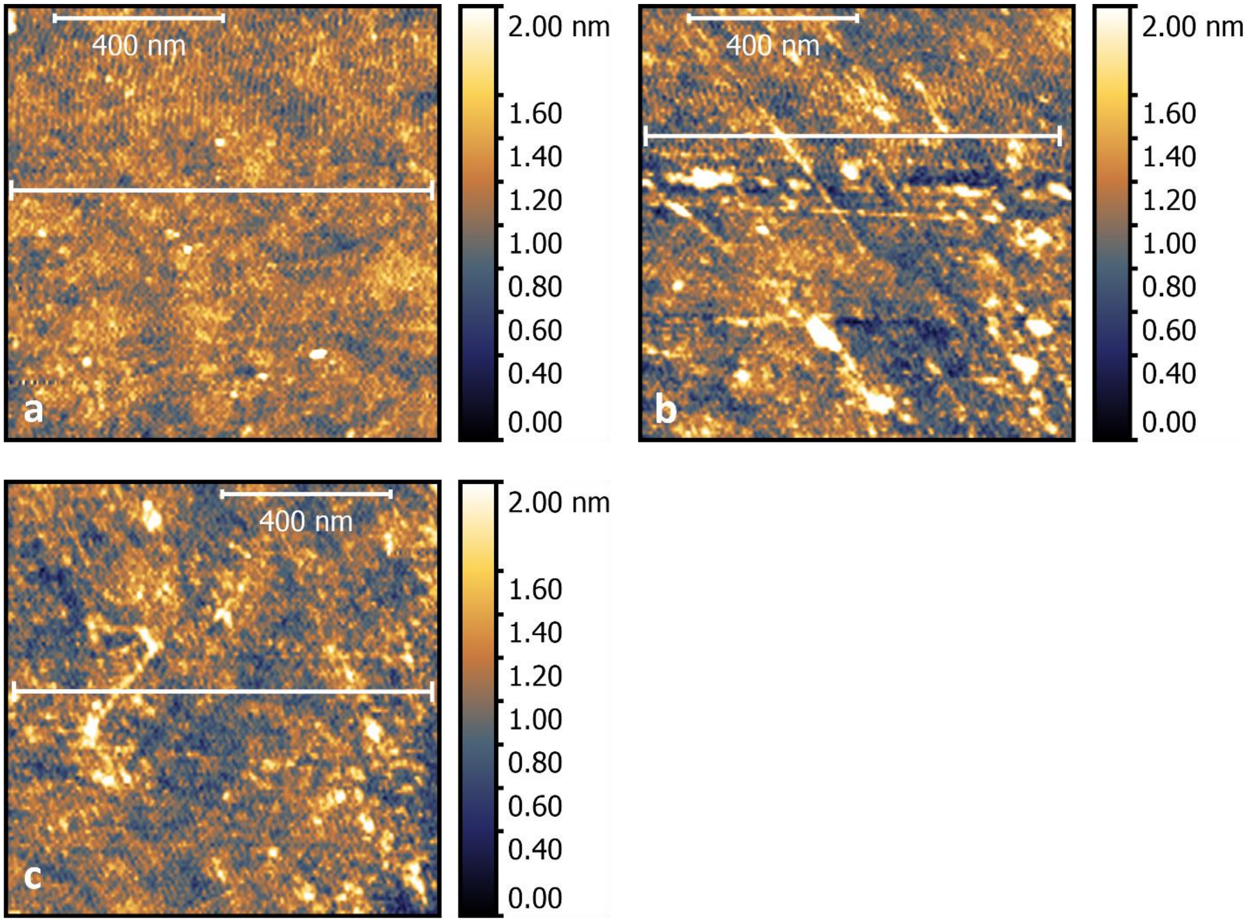

Supplementary Figure 17 | Atomic force microscopy of the surface of 2 nm-thick Pt. Representative atomic force microscopy images (top view) of sample with 2 nm-thick Pt channel.

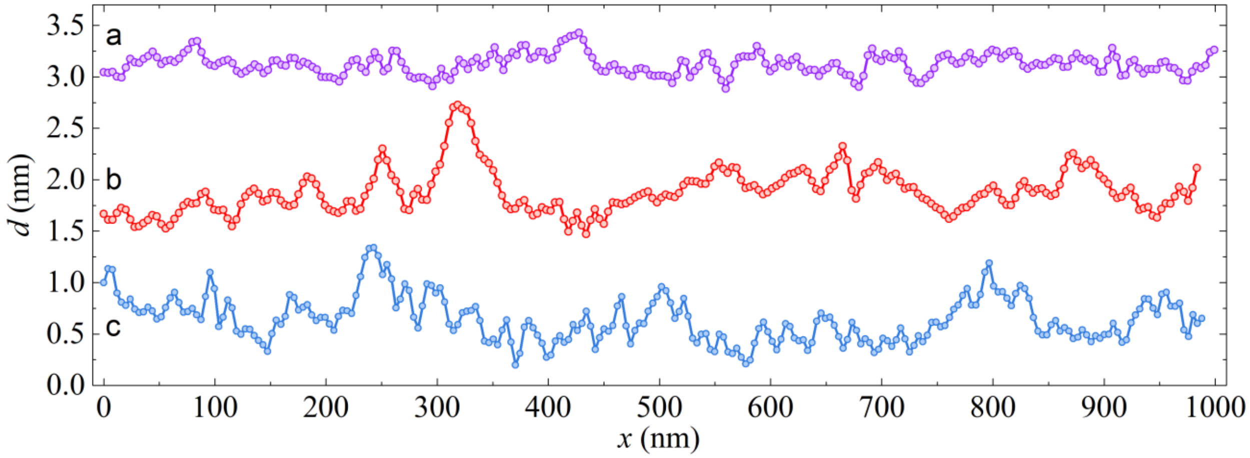

Supplementary Figure 18 | Atomic force microscopy profiles of 2 nm-thick Pt. Profile cuts of the atomic force microscopy images of sample with 2 nm-thick Pt channel denoted by the white lines in Supplementary Figure 17: purple filled circles – Supplementary Figure S17a, red filled circles – Supplementary Figure S17b, blue filled circles – Supplementary Figure S17c. Curves are shown with the offset along the *y*-axis for clarity.

Supplementary Note 12. Hall effect and magnetoresistance measurements

Hall effect and magnetoresistance measurements were performed on 2 nm-thick Pt sample using Physical Property Measurement System (PPMS - Quantum Design, USA). Supplementary Figure 19 shows in-plane electromotive force V_{xy} measured in the direction perpendicular to the drain-source current $I_{DS} = I_{xx} = 100$ μA under the upsweep (from negative to positive values, where positive field enters Pt from the YIG layer) of out-of-plane external magnetic field. We observed clear non-linear component that can be attributed to the anomalous Hall effect only at 10 K. Moreover, the negative magnetoresistance in Pt was also attributed to the emergence of the magnetic moments.[4] Supplementary Figure 20 shows temperature dependence of the in-plane electromotive force V_{xx} measured in the direction of the applied the drain-source current ($I_{DS} = I_{xx}$ = 100 μA) under the upsweep of out-of-plane external magnetic field.. We observe switching from the positive to the negative magnetoresistance only at 10 K. Thus, magnetic effects appear in our system at much lower temperature than the 250 K at which spin pumping and spin-charge conversion experiments were performed. Spike-like noise near the zero magnetic field is probably caused by the stray magnetic fields, and will be investigated in detail elsewhere.

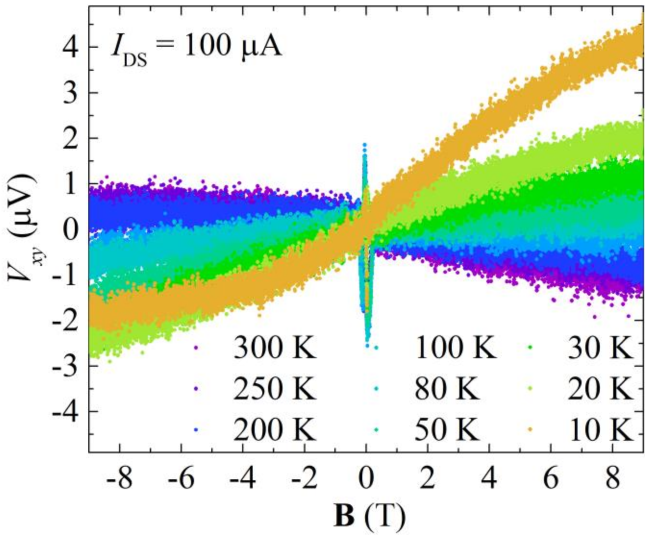

Supplementary Figure 19 | Temperature dependence of Hall voltage of 2 nm-thick Pt. Hall voltage measured from the sample with 2 nm-thick Pt channel in the temperature range 10 K – 300 K under the upsweep of out-of-plane magnetic field.

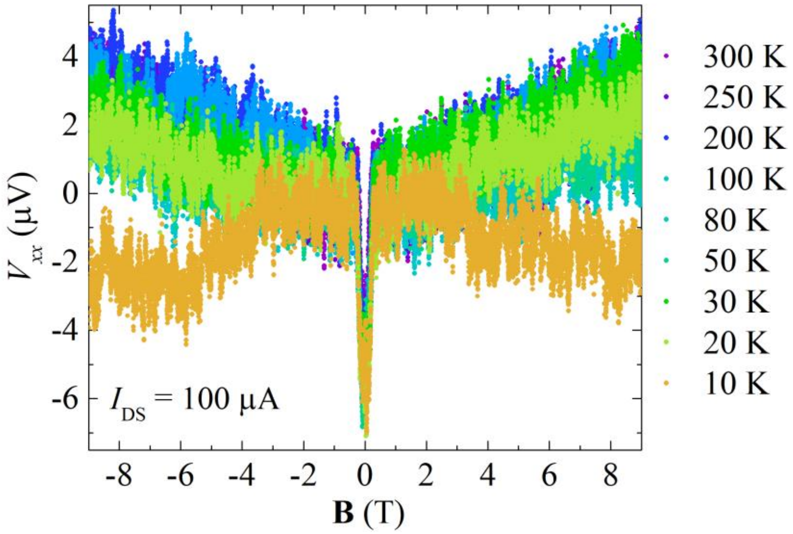

Supplementary Figure 20 | Temperature dependence of magnetoresistance voltage of 2 nm-thick Pt. Magnetoresistance voltage measured from the sample with 2 nm-thick Pt channel in the temperature range 10 K – 300 K under the upsweep of out-of-plane magnetic field.

Supplementary Note 13. Gate dependence of the Gilbert damping parameter

Supplementary Figure 21 shows the Gilbert damping parameter dependence on the gate voltage application for 2 nm-thick Pt (Device A in Fig. 4 and 5 of the Main Text). Each filled circle corresponds to the distinct value of the applied V_G, and the x-axis shows the resistivity of the sample measured at the applied V_G. y-axis shows the Gilbert damping parameter extracted from the microwave absorption spectrum (Supplementary Note 8) for the direction of the external magnetic field $\theta_H = 0°$ (purple filled circles) and $\theta_H = 180°$ (blue filled circles). The dependence of the Gilbert damping parameter on the resistivity of the underlying layer is given by[17]

$$\alpha = \alpha_0 + \alpha_{sp} = \alpha_0 + g^{\uparrow\downarrow} \frac{g\mu_B}{4\pi|\mathbf{M}|d_{YIG}} \left(1 + g^{\uparrow\downarrow}\rho_{Pt} \frac{2e^2\lambda_s}{h\tanh(d_{Pt}/\lambda_s)}\right)^{-1}, \quad (3)$$

where α_0 is the Gilbert damping parameter of YIG film without spin pumping contribution, α_{sp}—spin pumping contribution to the Gilbert damping, $g^{\uparrow\downarrow}$—real part of the spin-mixing conductance, g = 2.046—g-factor of the YIG layer,[18] μ_B—Bohr magneton, $4\pi|\mathbf{M}|$ = 1830 G—effective magnetization of the YIG layer determined from the experimentally measured ferromagnetic resonance using Kittel equation, d_{YIG} = 1300 nm—thickness of the YIG layer, d_{Pt} = 2 nm—thickness of the Pt layer, ρ_{Pt}—resistivity of the Pt layer, e—elementary charge, λ_s—spin diffusion length, h—Planck constant. The coefficient β that links the spin diffusion length to conductivity in Pt ($\lambda_s = \beta\cdot\sigma_{Pt}$) due to the Elliott-Yafet spin relaxation mechanism was reported to be in the range $\beta = (0.7\pm0.1)\cdot10^{-15}$ Ω m^2.[19–22] That gives the value of the spin diffusion length $\lambda_s = 0.5\pm0.1$ nm for d_{Pt} = 2 nm sample. Red line in Supplementary Figure 21 shows fitting of the experimentally measured Gilbert damping dependence on the resistivity (gate voltage application) using Supplementary Equation 3 and parameters above. The value of the spin mixing conductance obtained from the fitting $g^{\uparrow\downarrow} = (2.3\pm0.8)\cdot10^{19}$ m^{-2} is comparable to the values reported in the literature.[23] This is an effective value of the spin-mixing conductance, which can be affected by the spin flipping at the YIG/Pt interface.[19,24–26] We note that this estimation should be taken with care and only as an order of magnitude estimate: as one can see from the Supplementary Figure 21 change in the Gilbert damping parameter with application of gate voltage is comparable to the scatter in the experimental data. Spin pumping contribution to the Gilbert damping parameter is inversely proportional to the thickness of the YIG film.[27,28] Such dependence originates from the fact that total magnetic moment of the ferromagnet is proportional to its thickness, thus thin samples are more sensitive to the transfer of the angular momentum at the interface during the spin pumping. For example, enhancement of the Gilbert damping parameter due to the spin pumping in Pt was measured to be $\alpha_{sp} = 2.1\cdot10^{-3}$ and $\alpha_{sp} = 3.6\cdot10^{-3}$ for 20 nm-thick YIG,[23,29] but only $\alpha_{sp} = 3\cdot10^{-4}$ for 96 nm-thick YIG.[30] Thus, for our YIG/Pt system with 1300 nm-thick YIG spin pumping contribution to the Gilbert damping parameter is expected to be $\alpha_{sp} \sim 2\cdot10^{-5}$ - $5\cdot10^{-5}$, which is the same order of magnitude as the scatter in the experimentally measured data in Supplementary Figure 21. We stress that this difficulty has fundamental origin in the large thickness of the YIG film, and the achieved precision of the measurement setup is already remarkably good. If one takes measurement error of the Gilbert damping parameter as $\Delta\alpha = 2\cdot10^{-5}$ (see Supplementary Figure

21), it corresponds to the Δ|**B**| = 0.007 mT error in the magnetic field measurement. Ferromagnetic resonance magnetic field of YIG in our setup (microwave frequency f = 9.12 GHz, in-plane external magnetic field geometry) is $|\mathbf{B}_{FMR}|$ ~ 240 mT. Thus, combined error of the electromagnet power source and Hall sensor for magnetic field measurement is only ~0.003%.

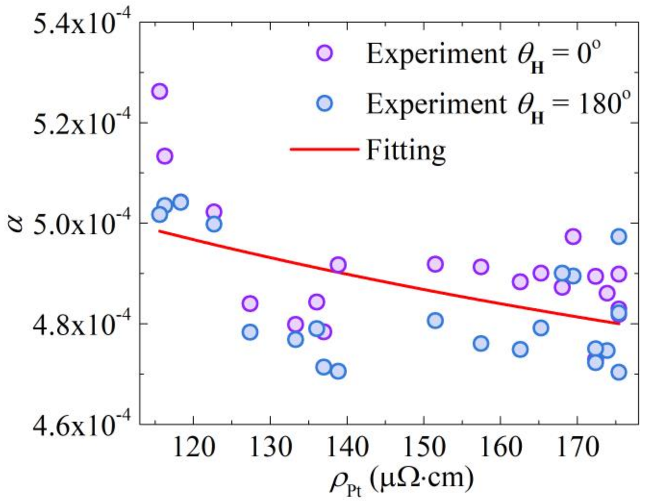

Supplementary Figure 21 | Dependence of the Gilbert damping parameter on Pt resistivity. Gilbert damping parameter dependence on the resistivity of the Pt film tuned by gate voltage application for 2 nm-thick Pt (Device A in Figs. 4 and 5 of the Main Text). Filled circles show experimentally measured Gilbert damping parameter extracted from the microwave absorption spectrum (Supplementary Note 8) for the direction of the external magnetic field $\theta_{\mathbf{H}}$ = 0° (purple filled circles) and $\theta_{\mathbf{H}}$ = 180° (blue filled circles). Red line shows fitting using Supplementary Equation 3.

Supplementary Note 14. Room temperature measurements of the ISHE modulation

Here, we comment on the possibility to exploit reported effect at room temperature. While we modulated ISHE at 250 K, it was due to the use of the ionic gel: the ionic leak current prevents accurate measurements at room temperature. The ISHE is known to be robust at room temperature, and in our devices without gate voltage application, we detected ISHE at both room and low temperatures. Thus, we believe that gate modulation of the ISHE in Pt can be successfully achieved at room temperature, with the room temperature-operational electric gate.

Supplementary Note 15. Gate modulation of the resistance and spin-charge conversion in the presence of the charge screening.

Any system with large carrier density has a short charge screening length, which limits the gate modulated region in the studied Pt samples to a few angstroms-thick layer. However, it does not prevent resistance of the sample being modulated by the gate. The calculated number of carriers and their effect on the resistance modulation of the samples (Fig. 3 of the main text) does not depend on the uniformity of the induced carriers, i.e. the resistance of the gated sample does not depend on the penetration depth of the electric field. The reason is that such system is equivalent to the combination of resistors connected in parallel. The top layer of Pt that is modulated by the gate application acts as a first resistor, while the rest of the channel—unaffected by the gate—acts as a second resistor. Since the two resistors are connected in parallel, gate modulation of the resistance of the first resistor affects the total resistance of the system. The total resistance of the sample would be determined by the number of the injected carries, and not by the screening length in the material. Thus, the resistance modulation can be observed for metallic film of any thickness, but the effect is very small for thick films: the resistance modulation under application of a specific gate voltage is controlled by the N_G/N_0 (the ratio of the injected number of carriers to number of carriers present in the sample), which is inversely proportional to the sample thickness d due to the relation $N_0=n_0 \cdot w \cdot l \cdot d$ (where l, w and n_0 are the length, width, and the internal carrier density in the channel, respectively). Such scaling of the resistance modulation described by the parallel resistors model was experimentally showed for Au, Ag, and Cu films with thicknesses up to 50 nm.[31,32]

Another important question is how the control over the top part of the 2 nm-thick Pt layer allows to effectively control the spin-charge conversion in the whole Pt layer. Due to the screening effect the electric field induced by the gate cannot reach the YIG/Pt interface, where the spin injection of the pure spin current happens. Hence, application of the gate voltage should not influence the spin pumping into the Pt from YIG. The important distinction here is that the spin injection via the spin pumping at YIG/Pt interface and the spin-charge conversion via the inverse spin Hall effect (ISHE) in Pt are two separate processes. While the first process cannot be controlled by the gate, the second process can be controlled. The spin-charge conversion happens on the characteristic scale of the spin diffusion length λ_s. The λ_s value in Pt can be estimated using the coefficient β that links the spin diffusion length to conductivity ($\lambda_s = \beta \cdot \sigma_{Pt}$) due to the Elliott-Yafet spin relaxation mechanism, where β = (0.7±0.1) fΩ·m^2 (value from the combined values reported in the literature: β = (0.61±0.02) fΩ m^2 ,[19] β = (0.63±0.02) fΩ·m^2,[20] β = (0.61±0.02) fΩ m^2,[21] β = (0.77±0.08) fΩ m^2 [22]).The estimated spin diffusion length λ_s = 0.5±0.1 nm for d_{Pt} = 2 nm sample. In our case, the 1 nm-thick Pt samples are not conductive, which is probably due to the surface roughness of the sample (difference in height of the Pt layer can be up to 1 nm as shown in AFM images in Supplementary Note 11), and additionally due to the thin oxidized/dead layer that might be present at the surface of the Pt film. Hence, the actual thickness d_{Pt} might be even smaller than the nominal value of 2 nm. It makes the spin diffusion length and thickness of the Pt film comparable in 2 nm-thick sample, and allows the injected spin current to reach the top Pt surface, where the gate is effective. Still, in such case, one might expect considerable part of the spin current converted into a charge current before it reaches the gate-controlled part of Pt. That

would be true for thick Pt films, however, this is not the case in ultrathin films. The reason is the domination of the surface and grain boundaries scattering over the bulk scattering in thin films. As can be seen from the sharp increase in the resistance of Pt with decreasing thickness (Fig. 1 in the Main Text), the scattering in such thin films is controlled by the surface and grain boundary scattering events. The scaling for the spin diffusion length mentioned above ($\lambda_s = \beta \cdot \sigma_{Pt}$) provides the effective spin diffusion length: as if all the scattering events are evenly distributed through the Pt. The increase in the resistance due to the increased spatial density of the scattering events leads to the carrier experiencing the same number of the spin scattering events over the shorter path: leading to the shorter spin diffusion length. However, in ultrathin films most of the scattering events occur at the surface and grain boundaries: carrier needs to reach them to experience the spin scattering event. Since the grain size is roughly equal to the thickness in ultrathin films, after the injection of the pure out-of-plane spin current at YIG/Pt interface, the first grain boundary that carrier reaches is at the surface of the Pt film. Thus, the majority of the scattering events that govern the inverse spin Hall effect in ultrathin films occurs in the top part of the Pt layer, which is controlled by the gate. The resistance modulation, while small, can be observed even in thick Pt samples—the injected carriers affect Pt resistance at all thicknesses due to the parallel conduction channels. However, for the spin-charge conversion to be effectively controlled by the gate, the spin scattering events should mostly occur in the top Pt layer, which happens only in ultrathin Pt films, as explained above. Fig. 1 shows that contribution of the surface and grain boundaries scattering rapidly increases for $d_{Pt} < 5$ nm. That is why we cannot observe any modulation of the spin-charge conversion current in $d_{Pt} = 10$ nm samples (bulk scattering dominates), but we achieve complete control over the spin-charge conversion current in ultrathin $d_{Pt} = 2$ nm samples (surface and grain boundaries scattering dominates), as shown in Fig. 5c in the Main Text. Additionally, the smaller the thickness of the sample—the larger the part of the sample controlled by the gate—the easier it is for carriers to reach it without prior scattering.

Supplementary Note 16. Temperature dependence of the resistivity in ultrathin Pt films.

In this section we present data on the temperature dependence of the resistivity in ultrathin Pt films. Several mechanisms can contribute to the scattering (i.e. the resistivity) in the thin metallic films, however, they have very distinct temperature-dependent behaviors. The characteristic metallic behavior consists of the scattering due to the electron-phonon contribution and the residual resistivity due to the scattering by impurities, grain boundaries and surfaces. The electron-phonon term of the resistivity—described by the Bloch-Gruneisen theory—is linearly proportional to the temperature: $\rho_{el\text{-}ph} \propto T$, and switches to the $\rho_{el\text{-}ph} \propto T^5$ dependence only below the Debye temperature.[33–36] The residual resistivity term (scattering by impurities, grain boundaries and surfaces) increases with the decreasing film thickness, but is independent of temperature ρ_{res} = const, and basically acts as a baseline for the other terms contributing to resistivity.[36–40] In contrast, the resistivity due to the grain hopping exhibits opposite temperature behavior (resistivity increases with the decreasing temperature) with $\rho_{hp} \propto \exp(1/T^{1/4})$ [36,41] or $\rho_{hp} \propto \exp(1/T^{1/2})$.[42,43] Additionally, at very low temperatures electron-electron scattering contribution to the resistivity $\rho_{el\text{-}el} \propto T^2$ [44,45] and localization effects like Kondo effect with $\rho_{loc} \propto \ln(a/T)$ start to play a role.[46] Supplementary Figure 22 shows the measured temperature dependence of the resistivity of the 2 nm-thick YIG/Pt sample. At 250 K (temperature of the spin-charge conversion measurements) the resistivity exhibits metallic behavior. The resistivity in the range 100 K – 300 K exhibits pure linear behavior, and, hence, is dominated by the $\rho = \rho_{el\text{-}ph} + \rho_{res} = aT + \rho_{res}$, where a and ρ_{res} are constants (red line fitting in Supplementary Figure 22). From the fitting, the contributions to the resistivity of the sample at 250 K are estimated to be $\rho_{el\text{-}ph}$ = 13 μΩ cm and ρ_{res} = 133 μΩ cm. Thus, the grain boundaries and surface scattering term ρ_{res} provides the dominant contribution (91%) to the resistivity of the 2 nm-thick samples at the temperature of the spin-charge conversion measurements. This result is consistent with the previous studies[37–40] and measured in our study thickness dependence of the resistivity (Fig. 1 of the Main Text) that show large increase in the surface and grain scattering with decrease in the film thickness. At low temperatures we observed the minimum in the resistance at T = 27 K. The grain hopping mechanism and the Kondo effect could lead to the observed increase in the resistivity with the decreasing temperature. We fitted the resistivity in the 2 K – 40 K range—where the upturn and minimum in the resistivity was observed—using the fitting functions below.

Fitting function that includes resistivity term due to the grain hopping $\rho_{hp} \propto \exp(1/T^{1/4})$:

$$\rho_1^{hop} = \rho_{hp1} + \rho_{res} + \rho_{el\text{-}el} + \rho_{el\text{-}ph} = a \cdot \exp(1/T^{1/4}) + \rho_{res} + bT^2 + cT^5, \qquad (4)$$

where a, ρ_{res}, b, c are fitting parameters independent of temperature.

Fitting function that includes resistivity term due to the grain hopping $\rho_{hp} \propto \exp(1/T^{1/2})$:

$$\rho_2^{hop} = \rho_{hp2} + \rho_{res} + \rho_{el\text{-}el} + \rho_{el\text{-}ph} = a \cdot \exp(1/T^{1/2}) + \rho_{res} + bT^2 + cT^5, \qquad (5)$$

where (a, ρ_{res}, b, c are fitting parameters independent of temperature);

Fitting function that includes resistivity term due to the Kondo effect $\rho_{loc} \propto \ln(a/T)$:

$$\rho_3^{\text{Kondo}} = \rho_{\text{loc}} + \rho_{\text{res}} + \rho_{\text{el-el}} + \rho_{\text{el-ph}} = a_1 \cdot \ln(a_2/T) + \rho_{\text{res}} + bT^2 + cT^5 = a_1 \cdot \ln(a_2) - a_1 \cdot \ln(T) + \rho_{\text{res}} + bT^2 + cT^5 = -a_1 \cdot \ln(T) + \rho_{\text{res}}` + bT^2 + cT^5, \quad (6)$$

where a_1, ρ_{res}`, $b$, $c$ are fitting parameters independent of temperature, and $\rho_{\text{res}}` = a_1 \cdot \ln(a_2) + \rho_{\text{res}}$.

The fitting results are shown in Supplementary Figure 23. While based on the present data it is preliminary to attribute upturn in the resistivity to any single mechanism (Even taking into account that all samples exhibited linear *I-V* curves at all temperatures and applied gate voltages (see Supplementary Figures 10-13 and Supplementary Figure 24), which largely limits the extent to which any tunneling or hopping effects can be present in the samples. Also, as a side note, we observe the same minimum in the resistivity of the 2 nm-thick Pt films grown on top of the paramagnetic GGG substrates. Thus, it cannot originate from the Kondo effect due to possibly present at low temperatures magnetic moments in the YIG/Pt system discussed in Supplementary Note 12), we can estimate the upper bounds of these contributions at the spin-charge conversion measurements temperature of 250 K. When the rise in the resistance at low temperatures is attributed solely to the grain hopping effect, its contribution given by models $\rho_{\text{hp1}} \propto \exp(1/T^{1/4})$ (black line in Supplementary Figure 23) and $\rho_{\text{hp2}} \propto \exp(1/T^{1/2})$ (green line in Supplementary Figure 23) at 250 K is 5 and 4 μΩ cm, respectively, which is ≤ 3% of the total resistivity of the sample (146 μΩ cm). Thus, the upper bound of the decrease in the sample resistance due the gate control over the grain hopping mechanism (i.e. under the assumption that one can completely remove the resistivity contribution due to the grain hopping by the gate voltage application) is 3%. Experimentally, we observe decrease in the resistivity up to 53% in the 2 nm-thick samples at T = 250 K. Hence, change in the sample resistance under the gate voltage application cannot be attributed to the gate effect on the grain hopping scattering. In contrast, the estimation of the resistance modulation of Pt thin films due to the gate-induced carriers reproduces well the resistance change experimentally observed in our study. Finally, all samples exhibited linear *I-V* curves at all temperatures and applied gate voltages (see Supplementary Figures 10-13 and Supplementary Figure 24), which also indicates absence of any tunneling or hopping effects in our samples.

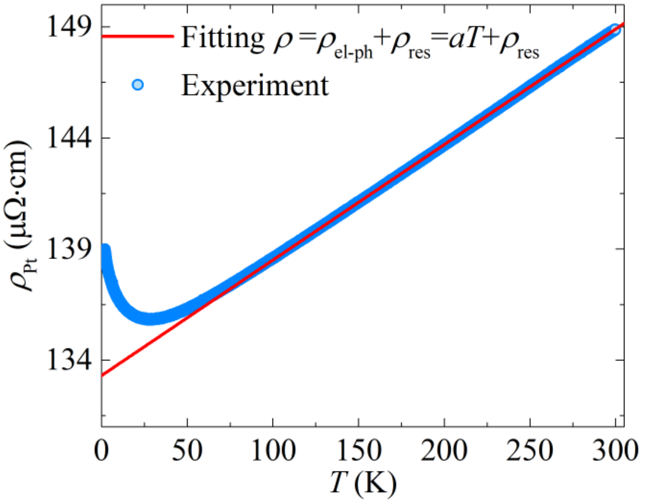

Supplementary Figure 22 | Temperature dependence of the resistivity of 2 nm-thick Pt. Blue filled circles—experimental data; red line shows fitting applied in the range from 120 K to 300 K using function $\rho = \rho_{el\text{-}ph} + \rho_{res} = aT + \rho_{res}$, where a and ρ_{res} are fitting parameters independent of temperature.

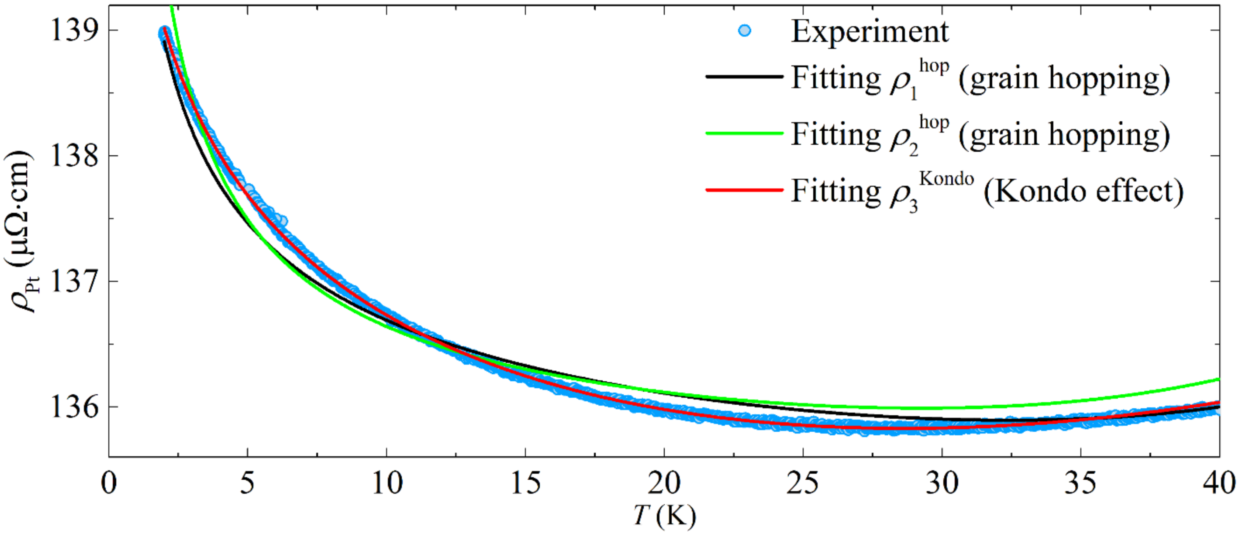

Supplementary Figure 23 | Temperature dependence of the resistivity of 2 nm-thick Pt. Blue filled circles—experimental data, fitting using ρ_1^{hop} with grain hopping term—black line, fitting using ρ_2^{hop} with grain hopping term—green line, fitting using ρ_3^{Kondo} with Kondo effect term—red line.

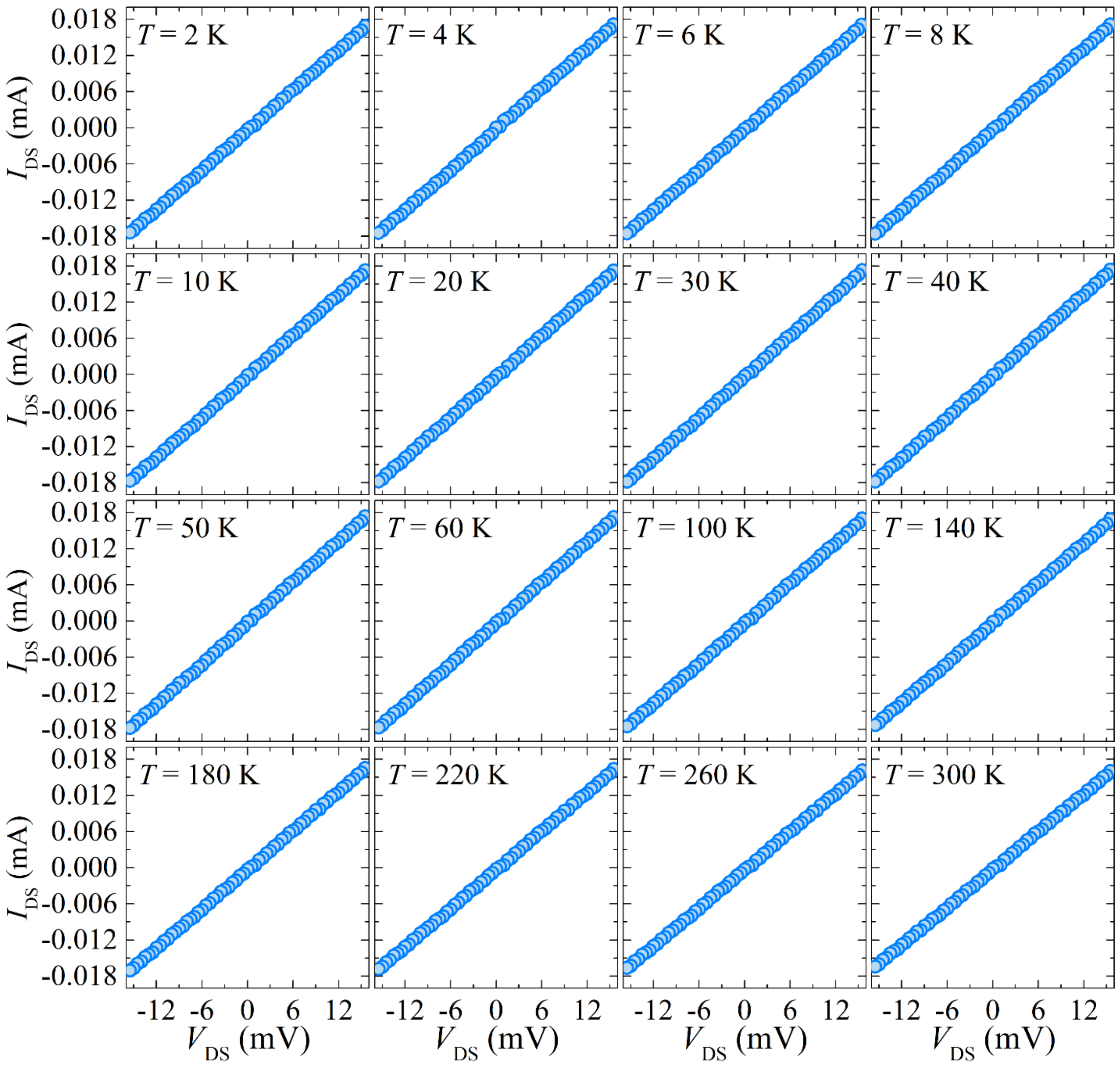

Supplementary Figure 24 | Temperature dependence of *I*-*V* characteristic of 2-nm thick Pt. Drain-source current I_{DS} dependence on the applied drain-source voltage V_{DS} at various temperatures for the 2 nm-thick Pt sample.

Supplementary Note 17. Amount of the impurities present in the Pt sputtering source.

Table 1 shows the amount of the impurities present in the Pt sputtering source, information is provided by the manufacturer (Tanaka Kikinzoku Kogyo Co., Ltd., Japan).

Element	Au	Ag	Pd	Rh	Ir	Ru	Os	Al	B	Bi	Ca	Cd	Co
Count (ppm)	ND	ND	10	20	12	6	ND	ND	4	ND	2	ND	ND

Element	Cr	Cu	Fe	Mg	Mn	Ni	Sb	Si	Sn	Ti	Zn	W	As
Count (ppm)	2	ND	11	ND	1	ND	ND	ND	ND	1	ND	ND	ND

Supplementary Table 1 | Impurity content of Pt sputtering source. Amount of the impurities present in the sputtering source that was used to grow Pt films. ND stands for “not detected”.

Supplementary References

1. Fan, P., Yi, K., Shao, J.-D. & Fan, Z.-X. Electrical transport in metallic films. *J. Appl. Phys.* **95,** 2527–2531 (2004).

2. Fischer, G. & Hoffmann, H. Size quantization of electronic states in very thin platinum films. *Zeitschrift Phys. B Condens. Matter* **39,** 287–297 (1980).

3. Petach, T. A., Lee, M., Davis, R. C., Mehta, A. & Goldhaber-Gordon, D. Mechanism for the large conductance modulation in electrolyte-gated thin gold films. *Phys. Rev. B* **90,** 081108 (2014).

4. Shimizu, S. *et al.* Electrically Tunable Anomalous Hall Effect in Pt Thin Films. *Phys. Rev. Lett.* **111,** 216803 (2013).

5. Sagmeister, M., Brossmann, U., Landgraf, S. & Würschum, R. Electrically Tunable Resistance of a Metal. *Phys. Rev. Lett.* **96,** 156601 (2006).

6. Aita, C. R. Optical behavior of sputter-deposited platinum-oxide films. *J. Appl. Phys.* **58,** 3169–3173 (1985).

7. Yang, Y., Sugino, O. & Ohno, T. Band gap of β-PtO 2 from first-principles. *AIP Adv.* **2,** 022172 (2012).

8. Zhensheng, J. Catalytic behavior of nanoparticle α-PtO2 for ethanol oxidation. *J. Mol. Catal. A Chem.* **191,** 61–66 (2003).

9. Abe, Y., Yanagisawa, H. & Sasaki, K. Preparation of Oxygen-Containing Pt and Pt Oxide Thin Films by Reactive Sputtering and Their Characterization. *Jpn. J. Appl. Phys.* **37,** 4482–4486 (1998).

10. Hirose, S. *et al.* Impact of Electrode Oxidation on the Current Transport Properties at Platinum/(Niobium-Doped Strontium-Titanate) Schottky Junctions. *ECS J. Solid State Sci. Technol.* **3,** P243–P248 (2014).

11. McBride, J. R., Graham, G. W., Peters, C. R. & Weber, W. H. Growth and characterization of reactively sputtered thin-film platinum oxides. *J. Appl. Phys.* **69,** 1596–1604 (1991).

12. Nakayama, H. *et al.* Electroresistance Effect in Gold Thin Film Induced by Ionic-Liquid-Gated Electric Double Layer. *Appl. Phys. Express* **5,** 023002 (2012).

13. Dushenko, S., Ando, Y., Shinjo, T. & Shiraishi, M. Precise extraction of the Gilbert damping parameter from the ferromagnetic resonance spectrum with spin wave resonances. In preparation.

14. Motobayashi, K., Minami, K., Nishi, N., Sakka, T. & Osawa, M. Hysteresis of Potential-Dependent Changes in Ion Density and Structure of an Ionic Liquid on a Gold Electrode: In Situ Observation by Surface-Enhanced Infrared Absorption Spectroscopy. *J. Phys. Chem. Lett.* **4,** 3110–3114 (2013).

15. Uysal, A. *et al.* Structural Origins of Potential Dependent Hysteresis at the Electrified Graphene/Ionic Liquid Interface. *J. Phys. Chem. C* **118,** 569–574 (2014).

16. Uysal, A. *et al.* Interfacial ionic 'liquids': connecting static and dynamic structures. *J. Phys. Condens. Matter* **27,** 032101 (2015).

17. Tserkovnyak, Y., Brataas, A., Bauer, G. E. W. & Halperin, B. I. Nonlocal magnetization dynamics in ferromagnetic heterostructures. *Rev. Mod. Phys.* **77,** 1375–1421 (2005).

18. Castel, V., Vlietstra, N., van Wees, B. J. & Youssef, J. Ben. Frequency and power dependence of spin-current emission by spin pumping in a thin-film YIG/Pt system. *Phys. Rev. B* **86,** 134419 (2012).

19. Liu, Y., Yuan, Z., Wesselink, R. J. H., Starikov, A. A. & Kelly, P. J. Interface Enhancement of Gilbert Damping from First Principles. *Phys. Rev. Lett.* **113,** 207202 (2014).

20. Liu, Y. *et al.* Direct method for calculating temperature-dependent transport properties. *Phys. Rev. B* **91,** 220405 (2015).

21. Sagasta, E. *et al.* Tuning the spin Hall effect of Pt from the moderately dirty to the superclean regime. *Phys. Rev. B* **94,** 060412 (2016).

22. Nguyen, M. H., Ralph, D. C. & Buhrman, R. A. Spin Torque Study of the Spin Hall Conductivity and Spin Diffusion Length in Platinum Thin Films with Varying Resistivity. *Phys. Rev. Lett.* **116,** 126601 (2016).

23. Wang, H. L. *et al.* Scaling of Spin Hall Angle in 3d, 4d, and 5d Metals from Y3Fe5O12/Metal Spin Pumping. *Phys. Rev. Lett.* **112,** 197201 (2014).

24. Rojas-Sánchez, J.-C. *et al.* Spin Pumping and Inverse Spin Hall Effect in Platinum: The Essential Role of Spin-Memory Loss at Metallic Interfaces. *Phys. Rev. Lett.* **112,** 106602 (2014).

25. Jungfleisch, M. B., Lauer, V., Neb, R., Chumak, a V & Hillebrands, B. Improvement of the yttrium iron garnet/platinum interface for spin pumping-based applications. *Appl. Phys. Lett.* **103,** 022411 (2013).

26. Pütter, S. *et al.* Impact of the interface quality of Pt/YIG(111) hybrids on their spin Hall magnetoresistance. *Appl. Phys. Lett.* **110,** 012403 (2017).

27. Tserkovnyak, Y., Brataas, A. & Bauer, G. E. W. Enhanced Gilbert Damping in Thin Ferromagnetic Films. *Phys. Rev. Lett.* **88,** 117601 (2002).

28. Kapelrud, A. & Brataas, A. Spin Pumping and Enhanced Gilbert Damping in Thin Magnetic Insulator Films. *Phys. Rev. Lett.* **111,** 097602 (2013).

29. Du, C., Wang, H., Yang, F. & Hammel, P. C. Enhancement of Pure Spin Currents in Spin Pumping Y3Fe5O12/Cu/Metal Trilayers through Spin Conductance Matching. *Phys. Rev. Appl.* **1,** 044004 (2014).

30. Lustikova, J. *et al.* Spin current generation from sputtered Y3Fe5O12 films. *J. Appl. Phys.* **116,** 153902 (2014).

31. Daghero, D. *et al.* Large Conductance Modulation of Gold Thin Films by Huge Charge Injection via Electrochemical Gating. *Phys. Rev. Lett.* **108,** 066807 (2012).

32. Tortello, M. *et al.* Huge field-effect surface charge injection and conductance modulation in metallic thin films by electrochemical gating. *Appl. Surf. Sci.* **269,** 17–22 (2013).

33. Bloch, F. Zum elektrischen Widerstandsgesetz bei tiefen Temperaturen. *Zeitschrift Phys.* **59,** 208–214 (1930).

34. Grüneisen, E. Die Abhängigkeit des elektrischen Widerstandes reiner Metalle von der Temperatur. *Ann. Phys.* **408,** 530–540 (1933).

35. Allen, P. B. & Butler, W. H. Electrical conduction in metals. *Phys. Today* **31,** 44–49 (1978).

36. *Springer Handbook of Electronic and Photonic Materials*. (Springer International Publishing, 2017). doi:10.1007/978-3-319-48933-9

37. Tanner, D. B. & Larson, D. C. Electrical Resistivity of Silver Films. *Phys. Rev.* **166,** 652–655 (1968).

38. Bid, A., Bora, A. & Raychaudhuri, A. K. Temperature dependence of the resistance of metallic nanowires of diameter $\geqslant$ 15 nm: Applicability of Bloch-Gruneisen theorem. *Phys. Rev. B* **74,** 035426 (2006).

39. Satrapinski, A., Hahtela, O. M., Savin, A. M., Novikov, S. & Lebedeva, N. Temperature Dependence of Pd Thin-Film Cryoresistors. *IEEE Trans. Instrum. Meas.* **60,** 2469–2474 (2011).

40. Timalsina, Y. P. *et al.* Effects of nanoscale surface roughness on the resistivity of ultrathin epitaxial copper films. *Nanotechnology* **26,** 075704 (2015).

41. Mott, N. F. Conduction in non-crystalline systems. *Philos. Mag.* **17,** 1259–1268 (1968).

42. Sheng, P., Abeles, B. & Arie, Y. Hopping Conductivity in Granular Metals. *Phys. Rev. Lett.* **31,** 44–47 (1973).

43. Simanek. E. The temperature dependence of the electrical resistivity of granular metals. *Solid State Commun.* **40,** 1021–1023 (1981).

44. Landau, L. D. & Pomeranchuk, I. On the Properties of Metals at Very Low Temperatures. *Zh. Eksp. Theo. Fiz.* **7,** 379 (1937).

45. Baber, W. G. The Contribution to the Electrical Resistance of Metals from Collisions between Electrons. *Proc. R. Soc. A Math. Phys. Eng. Sci.* **158,** 383–396 (1937).

46. Kondo, J. Resistance Minimum in Dilute Magnetic Alloys. *Prog. Theor. Phys.* **32,** 37–49 (1964).
